# Longitudinal testing of hippocampal plasticity reveals the onset and maintenance of endogenous human Aß-induced synaptic dysfunction in individual freely behaving pre-plaque transgenic rats: rapid reversal by anti-Aß agents

**DOI:** 10.1186/s40478-014-0175-x

**Published:** 2014-12-24

**Authors:** Yingjie Qi, Igor Klyubin, Sarah C Harney, NengWei Hu, William K Cullen, Marianne K Grant, Julia Steffen, Edward N Wilson, Sonia Do Carmo, Stefan Remy, Martin Fuhrmann, Karen H Ashe, A Claudio Cuello, Michael J Rowan

**Affiliations:** Department of Pharmacology and Therapeutics, Watts Building, Trinity College, Dublin 2, Ireland; Institute of Neuroscience, Trinity College, Dublin 2, Ireland; Department of Physiology, Trinity College, Dublin 2, Ireland; N. Bud Grossman Centre for Memory Research and Care, University of Minnesota, Minneapolis, MN 55455 USA; Department of Neurology, University of Minnesota, Minneapolis, MN 55455 USA; Institute for Translational Neuroscience, University of Minnesota, Minneapolis, MN 55455 USA; Geriatric Research Education Clinical Centre, VA Medical Centre, Minneapolis, MN 55417 USA; German Center for Neurodegenerative Diseases (DZNE), 53175 Bonn, Germany; Department of Pharmacology and Therapeutics, McGill University, 3655 Sir-William-Osler Promenade, Room 1210, Montreal, QC H3G1Y6 Canada; Department of Anatomy and Cell Biology, McGill University, Montreal, H3G1Y6 Canada; Department of Neurology and Neurosurgery, McGill University, Montreal, H3G1Y6 Canada

**Keywords:** Alzheimer’s disease, Amyloid ß, Transgenic rat, Long-term potentiation (LTP), Secretase inhibitor, Immunotherapy, Longitudinal

## Abstract

Long before synaptic loss occurs in Alzheimer’s disease significant harbingers of disease may be detected at the functional level. Here we examined if synaptic long-term potentiation is selectively disrupted prior to extracellular deposition of Aß in a very complete model of Alzheimer’s disease amyloidosis, the McGill-R-Thy1-APP transgenic rat. Longitudinal studies in freely behaving animals revealed an age-dependent, relatively rapid-onset and persistent inhibition of long-term potentiation without a change in baseline synaptic transmission in the CA1 area of the hippocampus. Thus the ability of a standard 200 Hz conditioning protocol to induce significant NMDA receptor-dependent short- and long-term potentiation was lost at about 3.5 months of age and this deficit persisted for at least another 2–3 months, when plaques start to appear. Consistent with *in vitro* evidence for a causal role of a selective reduction in NMDA receptor-mediated synaptic currents, the deficit in synaptic plasticity *in vivo* was associated with a reduction in the synaptic burst response to the conditioning stimulation and was overcome using stronger 400 Hz stimulation. Moreover, intracerebroventricular treatment for 3 days with an N-terminally directed monoclonal anti- human Aß antibody, McSA1, transiently reversed the impairment of synaptic plasticity. Similar brief treatment with the BACE1 inhibitor LY2886721 or the γ-secretase inhibitor MRK-560 was found to have a comparable short-lived ameliorative effect when tracked in individual rats. These findings provide strong evidence that endogenously generated human Aß selectively disrupts the induction of long-term potentiation in a manner that enables potential therapeutic options to be assessed longitudinally at the pre-plaque stage of Alzheimer’s disease amyloidosis.

## Introduction

The detection and characterization of the prodromal phase of Alzheimer’s disease (AD) has become a major focus of research and potentially provides the opportunity to intervene before clinical symptoms manifest [1]. Functional synaptic deficits mediated by pathogenic Aß in vulnerable pathways may provide a means of developing much-needed therapeutic avenues [2].

There is compelling evidence that acute exogenous application of soluble Aß aggregates, including oligomer-containing media from AD brain extracts, strongly and selectively disrupt hippocampal synaptic plasticity in brain slices *in vitro* and in anaesthetized animals *in vivo*, inhibiting the induction of long-term potentiation (LTP) without affecting baseline synaptic transmission [2,3]. The onset of the synaptic plasticity deficit is very rapid, occurring within minutes, but recently we found that the inhibition of LTP induction can persist for more than a week after injection of soluble AD brain Aß [4].

Research addressing the question as to whether or not endogenously generated Aß affects LTP in a manner similar to that caused by exogenous Aß has yielded mixed findings [5,6]. Although LTP has been widely reported to be impaired in many human amyloid precursor protein (APP)-based transgenic (TG) mouse models the selectivity of the deficit relative to reductions in baseline synaptic transmission and the role of Aß remain uncertain.

The investigation of non-murine animal models of AD amyloidosis offers new means of exploring early disease pathophysiology and thereby more rigorously testing the potential translational value of animal models. TG rats, in addition to introducing a species with many more human-like characteristics such as postnatal brain development and a more complex behavioural repertoire [7], offer many distinct practical advantages over mice for neurophysiological studies *in vivo*, particularly for long-term recordings in longitudinal designs. In particular, a novel, very complete, model of AD amyloidosis, the McGill-R-Thy1-APP TG rat provides an excellent means of exploring pre-plaque dysfunction [8-11] at the neurophysiological level.

Here we determined if, when and how synaptic plasticity is disrupted during the pre-plaque stage of amyloidosis in freely behaving and anaesthetized McGill-R-Thy1-APP TG rats *in vivo* and in hippocampal slices *in vitro*. We report a deficit in LTP induction that is associated with an impairment in synaptic burst responsiveness and NMDA receptor-mediated synaptic transmission. The synaptic plasticity deficit, tracked in chronically implanted individual rats, develops quickly over a critical period and remains stable for several months. Furthermore, brief treatment with agents targeting Aß via three different mechanisms rapidly, but transiently, restores the ability to induce LTP. The practical advantages of the rat for longitudinal neurophysiological investigation combined with the stable pre-plaque Aß-mediated deficit in LTP induction offers new opportunities to assess potential early interventions for AD *in vivo*.

## Materials and methods

### Animals

All experiments were carried out in accordance with guidelines under license from the Department of Health and Children, Ireland (86/609/EEC) using methods similar to those described previously [12]. Prior to surgery the animals were group-housed with free access to food and water and a 12-h lights on/off cycle. Male TG rats expressing human APP751 with Swedish and Indiana mutations under the control of the murine Thy1.2 promoter (McGill-R-Thy1-APP) [11] and their age-matched wild type (WT) littermates were studied. Animals were genotyped as outlined in Galeano et al. [8] and ages varied from 3 to 6 months old. Some in-house outbred 2–6 month-old Wistar rats, the background strain for the APP rats, were utilized in order to study the effects of D-AP5 and MRK-560 in WT animals.

### *In vivo* surgery and electrophysiology

For non-recovery experiments the rats were anaesthetized with urethane (1.5 g/kg, i.p.) and core body temperature was maintained at 37.5 ± 0.5°C. For recovery experiments the implantation procedure was comparable but carried out under anaesthesia using a mixture of ketamine and xylazine (80 and 8 mg/kg, respectively, i.p.) according to methods similar to those described previously [13]. For the recovery experiments the rats were allowed at least 14 days after surgery before recordings began. These rats were housed individually in their home cages post-surgery between recording sessions.

Teflon-coated tungsten wire (external diameter 75 μm bipolar or 112 μm monopolar) electrodes were positioned in the stratum radiatum of area CA1. Screw electrodes located over the contralateral cortex were used as reference and earth. The stimulation and recording electrodes were optimally located using a combination of physiological and stereotactic indicators. Field excitatory postsynaptic potentials (EPSPs) were recorded in the stratum radiatum of the dorsal hippocampus in response to stimulation of the ipsilateral Schaffer collateral-commissural pathway. The recording site was located 3.8 mm posterior to bregma and 2.5 mm lateral to midline, and the stimulating site was located 4.6 mm posterior to bregma and 3.8 mm lateral to midline. The final depths of the electrodes were adjusted to optimize the electrically evoked EPSP and confirmed by post-mortem analysis.

A stainless steel guide cannula (22 gauge, 0.7-mm outer diameter, length 13 mm) was implanted above the right lateral ventricle before the electrodes were implanted ipsilaterally. Injections were made via a Hamilton syringe which was connected to the internal cannula (28 gauge, 0.36 mm outer diameter). The injector was removed 1 min post-injection and a stainless steel plug was inserted. The position of the cannula was verified post-mortem by investigating the spread of ink dye after i.c.v. injection.

Test stimuli were delivered to the Schaffer-collateral/commissural pathway every 30 s to evoke field EPSPs that were 45-60% maximum amplitude. LTP was induced using our standard 200 Hz or a stronger 400 Hz high frequency stimulation (HFS) protocol. The 200 Hz protocol consisted of a single series of 10 trains of 20 stimuli with an inter-train interval of 2 s. The stimulation intensity was increased to 75% maximum for the anaesthetized rats. A repeated 400 Hz protocol (3 sets of 10 trains of 20 pulses, inter-train interval of 2 s and inter-set interval of 5 min), with the stimulation intensity increased to 75% maximum, was used to investigate NMDAR-independent LTP [14]. Paired-pulse facilitation (PPF) was measured as second/first EPSP amplitude ratio. The peak amplitude of the HFS-evoked field potential was expressed as a percentage the size of the test field EPSP evoked by single pulse stimulation.

Recovery animal experiments were carried out in a well-lit room. The recording compartment consisted of the base of the home cage, including normal bedding and food/water, but the sides were replaced with a translucent Perspex plastic box (27× 22× 30 cm) with an open roof. The rats had access to food and water throughout the whole recording session from the same position as in the home cage. All animals were first habituated to the recording procedure over the post-surgery recovery period.

### Slice preparation and electrophysiology

Rats were anesthetised with isoflurane, the brain removed and transverse hippocampal slices (350 μm) were prepared in ice-cold artificial cerebro-spinal fluid (ACSF) solution containing (in mM) 75 sucrose, 87 NaCl, 25 NaHCO_3_, 2.5 KCl, 1.25 NaH_2_PO_4_, 0.5 CaCl_2_, 7 MgCl_2_, 10 D-glucose, 1 ascorbic acid and 3 pyruvic acid. During incubation and experiments slices were perfused with ACSF containing (in mM) 125 NaCl, 25 NaHCO_3_, 2.5 KCl, 1.25 NaH_2_PO_4,_ 2 CaCl_2_, 1 MgSO_4_, 25 D-glucose. Slices were maintained at 33°C for 1 hr following dissection. All recordings were performed at physiological temperature (32–34°C).

Whole-cell patch-clamp recordings were made from CA1 pyramidal neurons, visualized using an upright microscope (Olympus BX51 WI, Middlesex, UK) with infra-red differential interference contrast optics (IR-DIC). Patch pipettes were pulled from thick-walled borosilicate glass (World Precision Instruments, Sarasota, FL) and had a resistance of 3–5 MΩ when filled with intracellular solution containing (in mM) 130 KMeSO_4_, 10 KCl, 0.2 EGTA, 10 HEPES, 20 phosphocreatine, 2 Mg_2_ATP, 0.3 NaGTP, 5 QX-314, (pH 7.3, 290–300 mOsm).

Unless otherwise stated, cells were voltage-clamped at −60 mV. Electrically evoked excitatory postsynaptic currents (EPSCs) were evoked at a test frequency of 0.033 Hz. Series resistance ranged from 5–17 MΩ and was compensated by 50–80% (5 kHz bandwidth). HFS consisted of 3 trains of 100 stimuli at 100 Hz, inter-train interval of 10 sec, delivered in current clamp mode. NMDAR-EPSCs were recorded in picrotoxin (100 μM), 2, 3 dioxo-6-nitro-1, 2, 3, 4-tetrahydrobenzo[f]quinoxaline-7-sulphonamide disodium salt (NBQX, 10 μM), CGP55845 (2 μM) and D-serine (20 μM) and using an intracellular solution with CsMeSO4 substituted for KMeSO4 and with the addition of QX-314 (5 μM) and TEA (1 mM). Recordings were made using a Multiclamp 700B (Molecular Devices, Foster City, CA). Signals were filtered at 5 kHz using a 4-pole Bessel filter and were digitized at 10 kHz using a Digidata 1440 analogue-digital interface (Molecular Devices).

Data were acquired and analyzed using PClamp 100, Clampfit (Molecular Devices) and Strathclyde Electrophysiology software (J. Dempster, University of Strathclyde, Glasgow, UK).

### Histology and immunohistochemistry

Rats were anaesthetized with an injection of ketamine and xylazine (260 and 10 mg/kg, respectively, i.p.). After transcardial perfusion with PBS pH 7.4 followed by 4% PFA for 15 min, brains were removed and fixed overnight in 4% PFA. Subsequently, the brains were stored in PBS with 0.01% sodium azide until cutting at 4°C. Hundred micrometer thick sections were cut on a vibratome (VT1200, Leica, Germany) and immunohistochemical staining was carried out. Free-floating sections were blocked for 30 minutes in 4% normal goat, 4% BSA and 0.4% Triton-X in PBS. An antibody detecting the N-terminus region of Aß in human APP (6E10) (mouse monoclonal, 1:1000, Covance Research Products Inc.) was applied to the solution and incubated overnight at room temperature. A counterstain was performed using the neuron-specific marker NeuN (1:1000, rabbit monoclonal, Merck Millipore). Sections were rinsed twice with PBS before secondary labeling. Anti-mouse secondary antibody AlexaFluor 488 and anti-rabbit secondary antibody AlexaFluor 647 (1:400, life technologies) were diluted in PBS and applied to the sections for two hours. Slices were mounted on coverslips using fluorescent mounting medium (Dako). Confocal microscopy was carried out on an inverted laser scanning microscope (LSM700, Zeiss) with a 10×NA0.3 air objective and 20×NA0.8 air objective. AlexaFluor 488 and 647 were excited at 488 nm and 633 nm respectively. The emission was detected with a SP 555 and LP650 filterset. Pictures were acquired with a resolution of 0.893 μm/pixel and 0.625 μm/pixel for zoom.

### Western blotting

Proteins from frontal cortex were extracted to yield fractions enriched in extracellular, intracellular, membrane-associated, and insoluble proteins as previously described [15]. Prior to biochemical analyses, all extracts were depleted of endogenous immunoglobulins by incubation with 50 μL Protein-G Fast Flow sepharose beads (GE Healthcare) for 1 h at 4°C. Samples were centrifuged at 9300 × g for 5 minutes to remove beads. Protein concentration was determined using a BCA protein assay kit (Thermo Scientific) according to the manufacturer’s instructions. 50 μg of membrane-associated extracts were used to measure APP expression. For immunoprecipitation experiments, 200 μg extracellular-enriched or 100 μg membrane-associated protein extracts were incubated with 5 μg 6E10 (Covance) and 50 μL Protein-G-coated magnetic beads (Life Technologies) overnight at 4°C. The beads were then sequentially washed for 20 min at 4°C with 1 mL buffer containing 50 mM Tris, 300 mM NaCl, 1 mM EDTA, and 0.1% Triton-X100 followed by 1 mL buffer containing 50 mM Tris, 150 mM NaCl, 1 mM EDTA, and 0.1% Triton-X100. In all cases, proteins were denatured by heating at 95°C for 5 min under denaturing conditions, separated by SDS-PAGE using 10-20% Tris-Tricine precast gels (Bio-Rad) and electro-transferred onto 0.2 μm nitrocellulose membranes at 0.4A for 3 h at 4°C. Membranes were boiled in 50 mL phosphate buffered saline by microwaving for 25 s, followed by a 4 min wait, then again for 15 s and blocked with 5% bovine serum albumin (Sigma-Aldrich) for 1 h at room temperature. Membranes were probed with anti-APPct (1:2500) (Invitrogen) followed by biotinylated donkey anti-rabbit secondary (1:60,000) (Jackson Laboratories) or probed with biotinylated 6E10 (1:2500). Membranes were then incubated with NeutrAvidin-HRP (1:5000) (Life Technologies). Signals were detected using West Pico electro-chemiluminescence (ECL) (Thermo Scientific) and bands were quantified using Optiquant software (Packard Instruments).

### Chemicals

McSA1 (Medimabs, Montreal, Canada) is a mouse monoclonal antibody that recognizes human Aß epitopes 1–12 [16]. The IgG control antibody 30B [17] was kindly provided by Dr Scott K. Dessain, Lankenau Institute of Medical Research, PA, USA).

The BACE1 (β–secretase 1) inhibitor LY2886721 (N-[3-[(4aS,7aS)-2-amino-4,4a,5,7-tetrahydrofuro[3,4-d][1,3]thiazin-7a-yl]-4-fluorophenyl]-5-fluoropyridine-2-carboxamide) (Axon Medchem, Groningen, The Netherlands) and γ-secretase inhibitor MRK-560 (*N*-[*cis*-4-[(4-Chlorophenyl)sulfonyl]-4-(2,5-difluorophenyl)cyclohexyl]-1,1,1-trifluoromethanesulfonamide) (Tocris, Bristol, UK) were initially dissolved in dimethyl sulfoxide (DMSO, Sigma, Dorset, UK). Final drug solutions were made up in sterile 0.9% saline or distilled water (total volume i.c.v. 5 μl/injection/rat). The NMDAR antagonists (R)-3-(2-carboxypiperazin-4-yl) propyl-1-phosphonic acid (CPP, Ascent Scientific) and D-AP5 (Tocris) were initially dissolved in distilled water. Control injections consisted of an equivalent amount of vehicle. CGP55845 was from Tocris (Bristol, UK). All salts used were obtained from Sigma as were picrotoxin and NBQX.

### Data analysis

The magnitude of potentiation is expressed as the percentage of baseline during the initial 10- (*in vitro*) or 30- (*in vivo*) min period, expressed as mean ± standard error of the mean, unless otherwise stated. For statistical analysis, EPSP and EPSC amplitudes were grouped into 5- (*in vitro*) or 10- (*in vivo*) min epochs. Standard one-way and two-way ANOVA, with repeated measures as appropriate, were used to compare between multiple groups followed by post hoc Bonferroni tests. Paired and unpaired Student's *t*-tests were used for 2-group comparisons. A P < 0.05 was considered statistically significant.

## Results

### Biochemical characterization of the pre-plaque stage in McGill-Thy1-APP-TG rats

**Figure 1:**
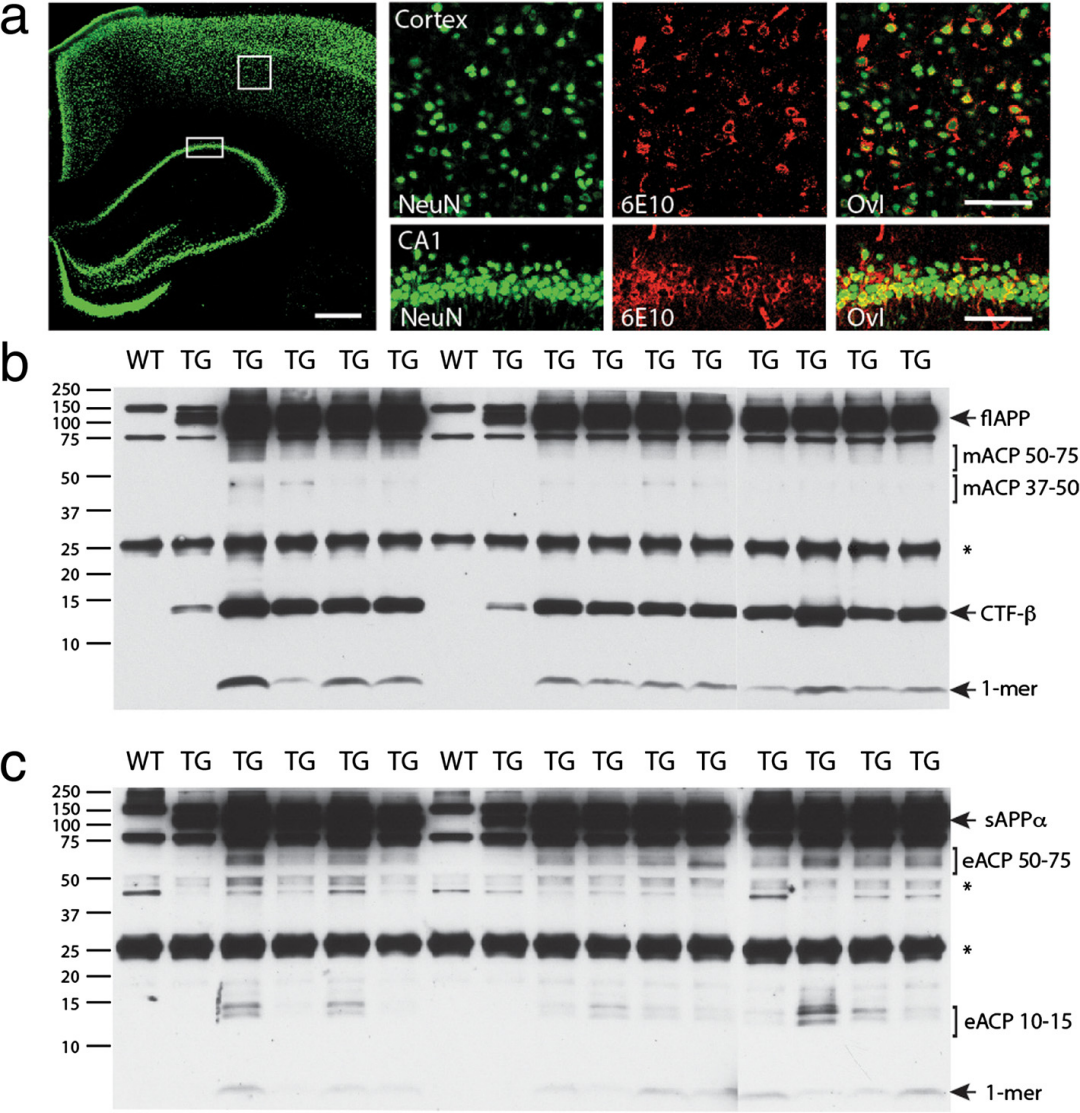
**Biochemical characterization of pre-plaque McGill-Thy1-APP-TG rat brain. (a)** Immunohistochemical staining with 6E10 (red), a monoclonal antibody to the N-terminal region of Aß in APP, illustrates the lack of extracellular deposits in the hippocampus of a 5 month-old TG rat. Sections were co-stained with the neuronal marker NeuN (green). Left hand panel shows low power view with NeuN staining. The boxed areas are shown in high power in the right hand panels. Several cell bodies had overlapping intracellular staining (yellow in overlay, Ovl) indicating intracellular reactivity in both hippocampal (CA1 area) and cortical neurons. The 6E10 staining that did not co-localize with NeuN staining was caused by non-specific staining of blood vessels. Scale bars: overview 500 μm, insets 100 μm. **(b,c)** Characterization by Western blotting using the antibody 6E10 of human APP breakdown products in membrane **(b)** and extracellular **(c)** fractions of cortical extracts from 3–4 month-old TG rats. **(b)** The membrane fraction contained Aß monomer (1-mer), C-terminal fragment-ß (CTF-ß) and full length APP (flAPP). It also contained two other APP cleavage products (mACP 50–75 and mACP 37–50). **(c)** In contrast, the extracellular fraction contained mainly sAPPα with trace amounts of Aß monomer and two other APP cleavage products (eACP 50–75 and eACP 10–15). The asterisks indicate non-specific bands.

Extracellular deposits of insoluble, fibrillar Aß only begin to be detectable in restricted brain areas, such as the subiculum, at ~7 months of age and mature dense plaques only become widespread across the cortex and hippocampus at ~12 months in McGill-Thy1-APP-TG rats [10,11] (and data not shown). Hippocampal and cortical neurons of 5 month-old pre-plaque TG animals show strong intracellular staining with the antibody 6E10, a human specific antibody directed to the N-terminus region of Aß in APP (Figure 1a). This staining is coherent with prior reports using a variety of antibodies, including some highly selective for Aß or oligomeric assemblies, that found evidence of the accumulation of non-fibrillar Aß aggregates in these brain regions from as early as 3 months of age [10,11]. Here we employed Western blotting (WB) techniques in order to determine if we could detect distinct Aß species in different brain fractions of these APP-overexpressing rats at 3–4 months of age. In the membrane-associated fraction of frontal cortex, the total level of APP expression was approximately 1.4 fold higher in TG rats than in WT littermates, as detected with an antibody that recognizes both human and rodent C-terminal APP (data not shown). Using 6E10, prominent bands corresponding to holo-APP along with C-terminal fragment-ß and Aß monomers were detected in the membrane fraction (Figure 1b). In contrast, in the extracellular-enriched cortical fraction from TG rats, whereas sAPPα was prominent, only faint amounts of Aß monomer were detected (Figure 1c). Thus, the membrane fraction, which includes intracellular membranes, contained considerably more Aß monomers than the extracellular fraction. Although we used highly sensitive techniques, that can readily detect SDS stable oligomeric aggregates in CSF and brain [18,19], we were unable to definitively ascribe any of the strong WB bands specifically to Aß oligomers in the 3–4 month-old TG rat brain fractions. It seems likely, given the compelling evidence for intracellular Aß aggregates at this age [10,11], that, compared to other APP fragments in a similar molecular weight range, the concentration of oligomers was relatively low in the membrane or extracellular fractions.

### Age-dependent pre-plaque inhibition of LTP in the CA1 area of freely behaving rats

**Figure 2:**
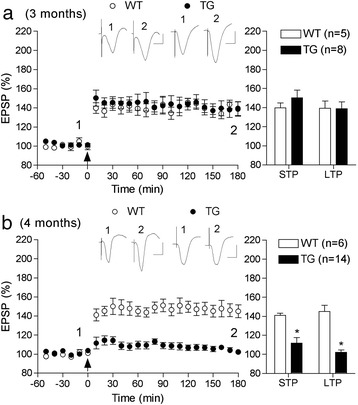
**Pre-plaque disruption of hippocampal LTP induction in freely behaving TG rats** ***in vivo*** **(a,b).** In freely behaving chronically implanted rats, LTP at CA3-to-CA1 synapses in the stratum radiatum was strongly impaired in 4 but not 3 month-old McGill-Thy1-APP-TG rats. **(a)** At 3 months of age application of a standard 200 Hz conditioning stimulation protocol (standard HFS, arrow) induced robust LTP that persisted for at least 3 h in both WT and TG rats. **(b)** In contrast, at 4 months of age, whereas the same conditioning stimulation triggered robust LTP in WT rats, the protocol only triggered a weak, non-significant, STP in TG rats. Time-course graphs are shown in the left hand panels with summary statistics for STP (first 10 min post-HFS) and LTP (last 10 min post-HFS) shown in the right hand panels. Insets show representative EPSP traces at the times indicated. Calibration bars: Vertical, 1.0 mV; horizontal, 10 ms. Values are the mean ± S.E.M. % pre-HFS baseline EPSP amplitude. *P < 0.05 compared with WT littermates.

In order to determine if hippocampal synaptic plasticity is disrupted before plaques are deposited in this TG rat line we attempted to induce LTP of excitatory glutamatergic transmission at CA3-to-CA1 synapses in the stratum radiatum of 3 and 4 month-old freely moving animals and their WT littermates. Animals were implanted with chronic electrode assemblies and acclimatized to the recording protocol in their home cage, which had been modified to allow wire attachment. We used our standard 200 Hz HFS protocol, which triggers an NMDA receptor-dependent LTP of synaptic transmission at these synapses [14,20,21]. In 3 month-old TG rats the application of HFS induced robust LTP that persisted for at least 3 h (138.8 ± 7.4% mean ± S.E.M. pre-HFS baseline, n = 8; P < 0.05 compared with baseline, two-way repeated measures ANOVA with post hoc Bonferroni test) and that was similar in magnitude to that induced in age-matched WT littermates (139.3 ± 7.6%, n = 5; P > 0.05 compared with TG; P < 0.05 compared with baseline) (Figure 2a). In marked contrast, LTP was strongly impaired in 4 month-old TG rats. Thus, at this age the 200 Hz conditioning protocol failed to induce LTP (102.2 ± 2.6%, n = 14; P > 0.05 compared with baseline; P < 0.05 compared with 145.0 ± 6.7% in 4 month-old WT littermates, n = 6) (Figure 2b). A similar significant reduction in the initial magnitude of potentiation (short-term potentiation, STP), as measured in the first 10 min after HFS, was present at 4 months of age in TG rats (111.6 ± 6.2%; P < 0.05 compared with 141.0 ± 2.2% in WT; P > 0.05 compared with baseline) (Figure 2b).

This age-dependent deficit in the ability to induce STP and LTP in TG rats appeared to be relatively selective since baseline AMPA receptor-mediated synaptic excitability, as measured by input–output properties, and PPF, measured using a 40 ms inter-pulse interval (IPI), were not significantly different from WT controls at either 3 or 4 months of age (Table 1).

**Table 1:** **Baseline synaptic efficacy and paired pulse facilitation (PPF) were not significantly disrupted in freely behaving pre-plaque 3–6 month-old McGill-Thy1-APP TG rats**

	**Age (month)**	**WT**	**n**	**TG**	**n**
**Baseline EPSP**	3	1.5 ± 0.2	(5)	1.5 ± 0.6	(7)
**(50%** **maximum amplitude, mV)**	4	1.2 ± 0.2	(5)	1.2 ± 0.1	(6)
	6	1.0 ± 0.3	(5)	0.9 ± 0.3	(6)
**Stimulation intensity**	3	9.2 ± 0.3	(5)	9.4 ± 0.2	(7)
**(50%** **maximum, mA)**	4	9.8 ± 0.2	(5)	9.9 ± 0.3	(6)
	6	10.8 ± 0.6	(5)	10.7 ± 0.3	(6)
**Stimulation intensity**	3	13.3 ± 0.3	(5)	12.9 ± 0.4	(7)
**(maximum, mA)**	4	12.2 ± 0.4	(5)	12.0 ± 0.6	(6)
	6	12.8 ± 0.6	(5)	13.1 ± 0.6	(6)
**PPF (ratio)**	3	1.6 ± 0.1	(5)	1.6 ± 0.1	(7)
**40 ms IPI**	4	1.6 ± 0.1	(5)	1.6 ± 0.1	(6)
	6	1.5 ± 0.1	(5)	1.5 ± 0.1	(6)

### Increasing the conditioning stimulation strength overcomes the inhibition of LTP

**Figure 3:**
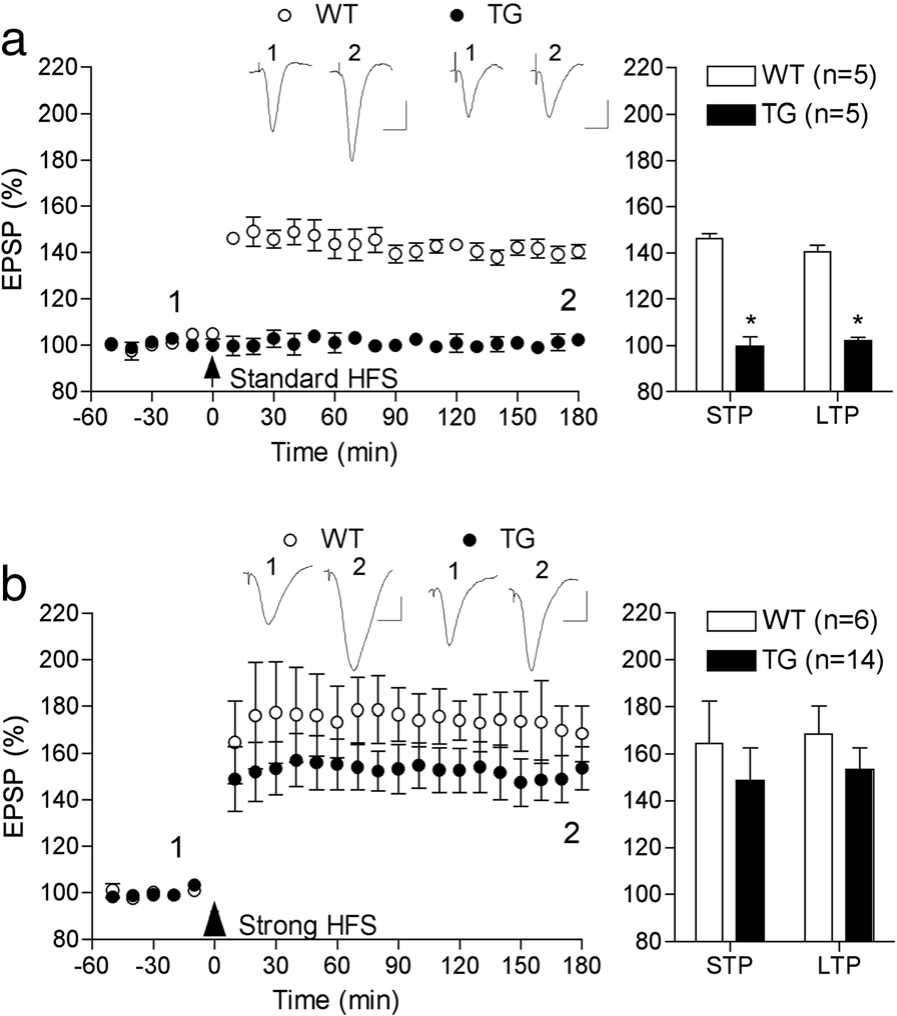
**Increasing the strength of the conditioning stimulation protocol overcame the inhibition of LTP in pre-plaque freely behaving TG rats. (a)** At 5–6 months of age the standard 200 Hz conditioning stimulation (arrow) triggered robust LTP in WT rats but failed to induce significant STP or LTP in TG rats. **(b)** Repeated high-intensity 400 Hz tetanization (strong HFS, large arrow head) in 5–6 month-old animals induced similar magnitude LTP in TG and WT rats. Time-course graphs are shown in the left hand panels with summary statistics for STP (first 10 min post-HFS) and LTP (last 10 min post-HFS) shown in the right hand panels. Insets show representative EPSP traces at the times indicated. Calibration bars: Vertical, 1.0 mV; horizontal, 10 ms. Values are the mean ± S.E.M. % pre-HFS baseline EPSP amplitude. *P < 0.05 compared with WT littermates.

Next we reasoned that if the inhibition of LTP in the TG rats was caused by an increase in the threshold for LTP induction, it should be possible to overcome the inhibition of LTP with a stronger conditioning stimulation protocol. For these studies we tested 5–6 month-old TG rats, an age when the standard HFS still failed to induce LTP (102.3 ± 1.3%, n = 5; P > 0.05 compared with baseline; P < 0.05 compared with 140.4 ± 3% in 5–6 month-old WT littermates, n = 5) (Figure 3a). The stronger conditioning protocol consisted of three sets of high-intensity, 400 Hz trains of stimuli, which triggers a large LTP in WT rats *in vivo* that is both NMDA receptor- and voltage-gated Ca^2+^ channel-dependent [14]. To our surprise, stimulation with this strong HFS protocol in pre-plaque, 5–6 month-old, TG rats induced robust LTP (153.5 ± 9.2% at 3 h post-HFS, n = 14; P < 0.05 compared with baseline), comparable in magnitude with that elicited in WT littermates (168.3 ± 11.8%, n = 6; P < 0.05 compared with baseline; P > 0.05 compared with TG group) (Figure 3b). Similar results were found for STP (148.8 ± 13.9 and 164.6 ± 17.8% in TG and WT, respectively) (Figure 3b)

.

Consistent with our previous findings in WT rats [14], the high-intensity 400 Hz protocol induced an LTP in the TG rats that was NMDA-receptor-dependent, being strongly blocked by pretreatment with the NMDA receptor antagonist CPP (10 mg/kg, i.p.) (118.4 ± 5.2%, n = 5; P < 0.05 compared with baseline; P < 0.05 compared with TG in the absence of CPP, data not shown).

### Inhibition of LTP and reduced synaptic burst responses in anaesthetized TG rats

**Figure 4:**
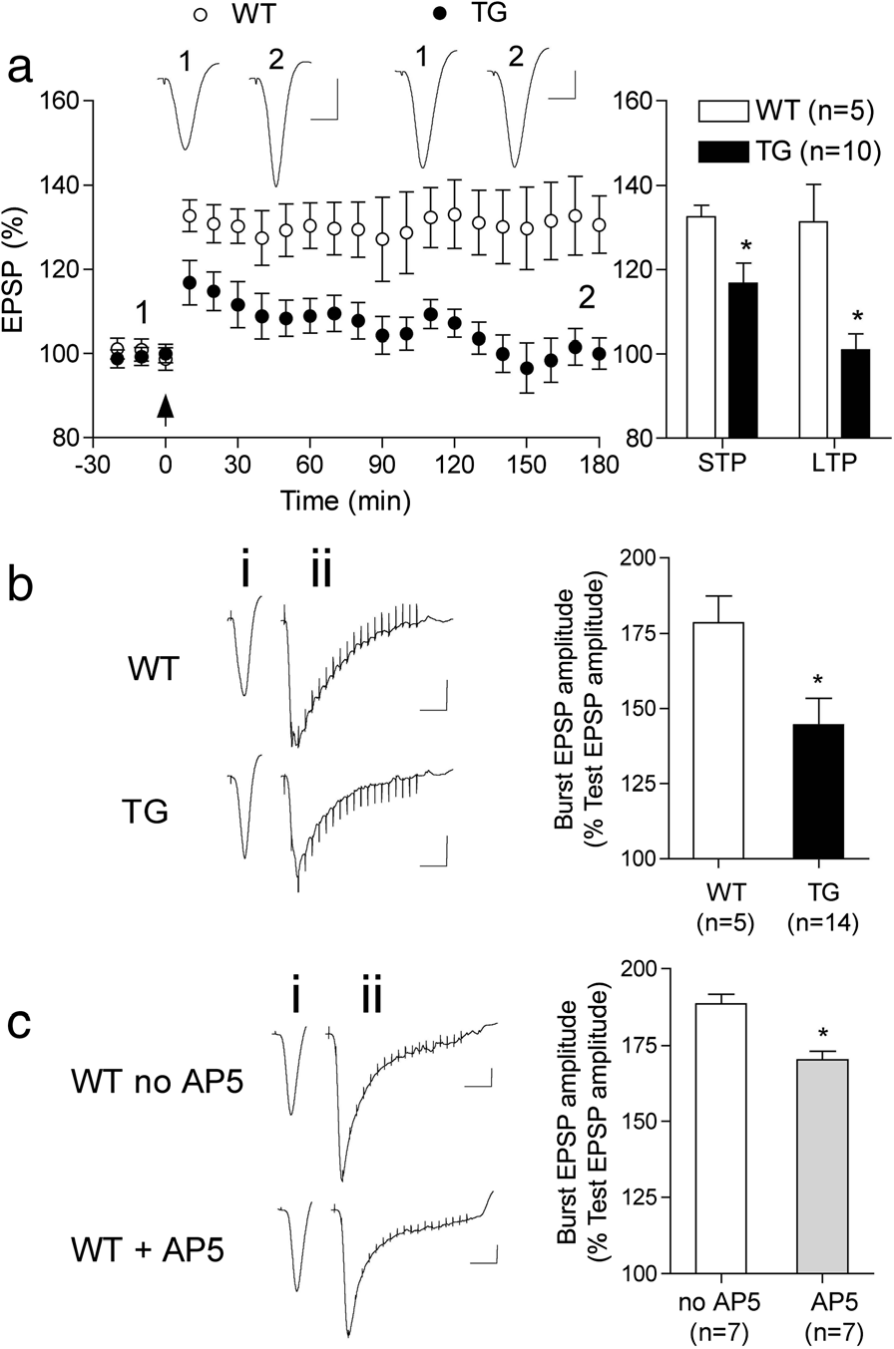
**LTP inhibition and reduced synaptic burst responses in anaesthetized TG rats. (a)** Both STP and LTP were strongly impaired in 3.5-4 month-old urethane anesthetized TG rats compared with age-matched WT rats. Standard 200 Hz conditioning stimulation (arrow) induced robust LTP in the controls but not in the TG rats. Time-course graphs are shown in the left hand panel. Insets show representative EPSP traces at the times indicated. Vertical, 1.0 mV; horizontal, 10 ms. **(b)** The peak amplitude of the HFS-evoked field potential (expressed as a % of the pre-HFS baseline EPSP amplitude) was significantly reduced in TG rats compared with WT littermates. Insets show representative EPSPs triggered by a single test pulse (i, left hand trace) and the average of 10 trains of 200 Hz burst of 20 stimuli (ii, right hand trace) in WT and TG rats. **(c)** The NMDA receptor antagonist D-AP5 reduced the peak amplitude of the HFS-evoked field potential in WT animals. Injection i.c.v. with 100 nmol D-AP5 10 min prior to HFS significantly reduced the burst response. Calibration bars: Vertical, 1.0 mV; horizontal, 20 ms. Values are the mean ± S.E.M. % pre-HFS baseline EPSP amplitude. Summary statistics are shown in the right hand panels. *P < 0.05 compared with WT littermates.

In order to determine if the deficit in LTP in the TG rats was relatively independent of behaviour, the ability to induce LTP with the standard 200 Hz conditioning stimulation protocol was evaluated in acutely anaesthetized 3.5-4 month-old animals. We chose this age because it was on the cusp of the age when the deficit in freely behaving animals was detected (see also longitudinal data in a later section of the Results section). Both STP and LTP were strongly impaired in the TG rats at this age. Thus HFS triggered a small STP and no significant LTP of excitatory synaptic transmission in these TG rats (116.7 ± 4.8 and 101.0 ± 3.6%, at 10 min and 3 h post-HFS, respectively, n = 10; P < 0.05 and P > 0.05 compared with baseline, respectively; P < 0.05 compared with 132.5 ± 2.7 and 131.3 ± 9.0%, respectively in WT littermates, n = 5) (Figure 4a).

There was no significant correlation between the magnitude of potentiation at either 10 min or 3 h post-HFS in the TG rats and the amount of Aß monomer in the membrane fraction of the cortical extracts measured by WB (Figure 1b and data not shown).

Similar to freely behaving animals, the deficit in the ability to induce LTP in anaesthetized TG rats appeared to be relatively selective since measures of baseline synaptic excitability or PPF (at 20, 40 and 100 ms IPI) were statistically indistinguishable between groups (Table 2). Although short-term plasticity, as measured by PPF, was not noticeably affected, the response to burst stimulation during the conditioning HFS protocol was attenuated in the TG rats. Thus the magnitude of the peak amplitude of the burst field potential response during the HFS was significantly smaller in TG rats (144.5 ± 8.8% single test EPSP amplitude, n = 14, compared with 178.5 ± 9.0%, n = 5, in WT littermates) (Figure 4b). Interestingly, there was a strong positive correlation between the magnitude of peak burst response to the HFS and the level of HFS-induced STP in the TG group (r = 0.62; P < 0.05). Since both synaptically generated bursting activity [22,23] and STP [24,25] are known to be NMDA receptor-dependent we tested the ability of the NMDA receptor antagonist D-AP5 to inhibit the burst response to HFS in WT animals under our recording conditions. Pretreatment with D-AP5 (100 nmol, i.c.v.) significantly reduced the peak burst response from 188.5 ± 3.2% to 170.2 ± 2.9% (n = 7; P < 0.05) (Figure 4c). These data are consistent with a deficit in LTP induction via an impairment of excitatory synaptic activation during the induction protocol.

**Table 2:** **Baseline synaptic efficacy and paired pulse facilitation (PPF) were not significantly disrupted in anaesthetized pre-plaque 3.5-4 month-old McGill-Thy1-APP TG rats**

	**WT** **(n = 5)**	**TG** **(n = 16)**
**Baseline EPSP**	2.0 ± 0.2	1.9 ± 0.1
**(50%** **maximum amplitude, mV)**		
**Stimulation intensity (mA)**		
50% max	6.2 ± 0.2	6.3 ± 0.2
75% max	8.0 ± 0.4	8.2 ± 0.2
100% max	13.4 ± 0.8	13.5 ± 0.3
**PPF (ratio)**		
20 ms IPI	1.6 ± 0.1	1.5 ± 0.05
40 ms IPI	1.7 ± 0.05	1.6 ± 0.1
100 ms IPI	1.5 ± 0.05	1.5 ± 0.05

### Inhibition of LTP and reduced NMDA receptor-mediated synaptic transmission *in vitro*

As described above, *in vivo* field potential measures of baseline excitatory synaptic transmission, which are AMPA receptor-mediated, were not significantly affected in TG rats at an age when NMDA receptor-dependent STP and LTP of that transmission was strongly impaired. Because the burst response to the conditioning stimulation in anaesthetized rats was reduced in a manner consistent with a decrease in NMDA receptor-mediated synaptic transmission, we were interested in examining glutamatergic mechanisms in TG rats at this age in detail. Therefore we carried out *in vitro* experiments that enabled us to directly measure AMPA and NMDA receptor-mediated components of synaptic transmission using intracellular recording methods. We monitored both electrically evoked and spontaneous currents at CA3-to-CA1 synapses from individual pyramidal cells using whole-cell patch clamp techniques in hippocampal slices.

**Figure 5:**
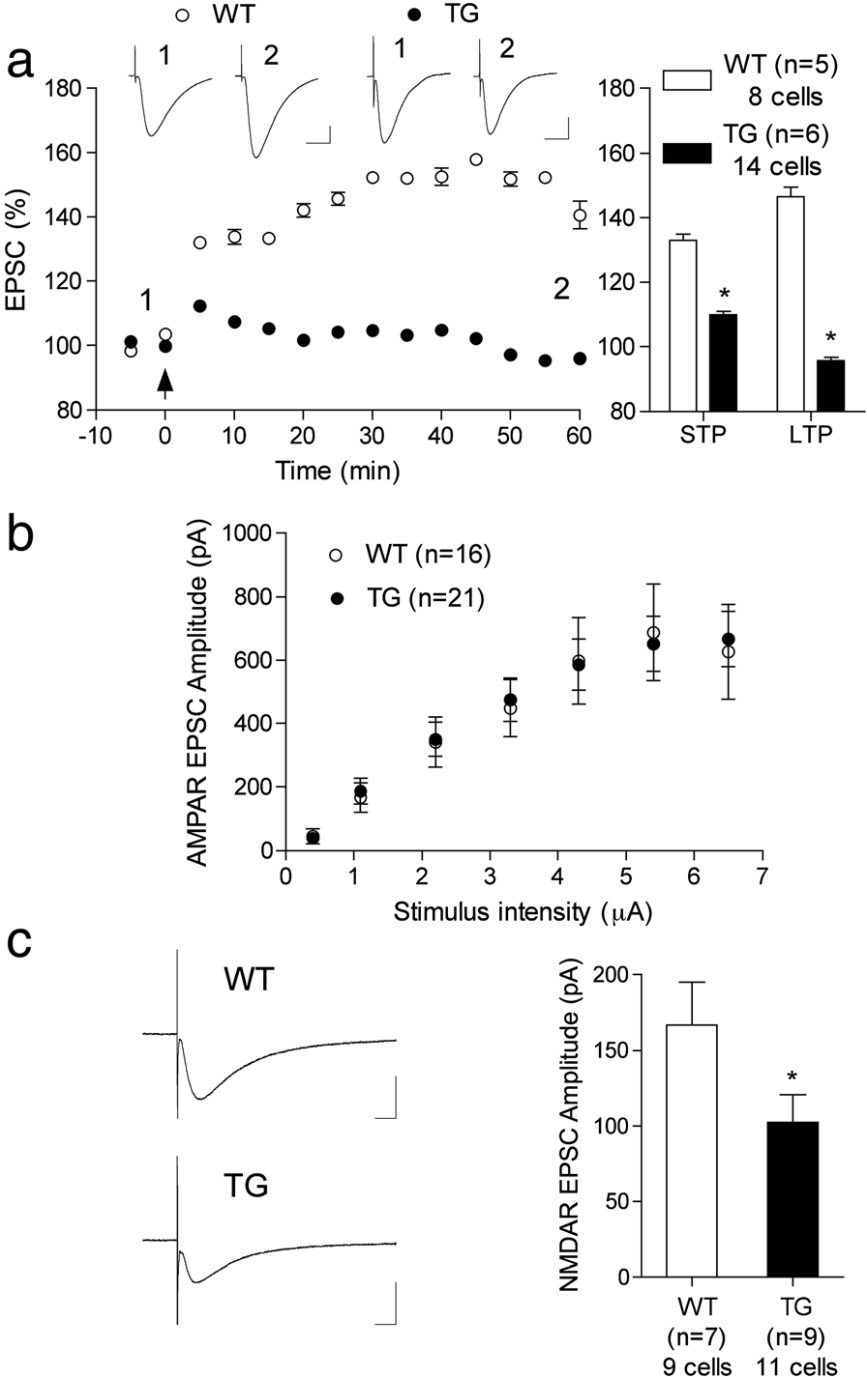
**LTP inhibition and reduced NMDA receptor-mediated synaptic transmission in TG rat hippocampal slices** ***in vitro.*** **(a)** LTP was strongly impaired in hippocampal slices from 3.5-4 month-old TG rats. The magnitude of potentiation at 5 (STP) and 60 (LTP) min after application of a 100 Hz conditioning stimulation protocol (arrow) was significantly reduced in TG animals compared with their WT littermates. **(b,c)** Whereas baseline AMPA receptor-mediated synaptic currents were not affected, NMDA receptor-mediated synaptic currents were reduced. **(b)** The input/output relationship for AMPA receptor-mediated EPSCs was very similar in WT and TG rats. **(c)** In contrast, NMDA receptor-mediated EPSCs were significantly smaller in TG rats. Insets show representative EPSC traces. Calibration bars: Vertical, 100 pA; horizontal, 10 ms. Values are the mean ± S.E.M. % pre-HFS baseline EPSC amplitude. Summary statistics are shown in the right hand panels. *P < 0.05 compared with WT littermates.

First, we assessed the ability of a standard set of 1 s trains at 100 Hz to induce NMDA receptor-dependent LTP [25,26]. Consistent with the *in vivo* field EPSP findings described above, both STP and LTP of electrically evoked AMPA receptor-mediated synaptic currents were strongly impaired in slices from 3.5-4 month-old TG rats. Whereas the 100 Hz conditioning protocol triggered robust STP and LTP in WT littermate controls (132.1 ± 1.5 and 140.6 ± 4.3% at 5 and 60 min post-HFS, respectively; P < 0.05 compared with baseline), this was not the case in TG rats (112.3 ± 1.5 and 96.1 ± 1.6%, respectively; P < 0.05 compared with the WT group) (Figure 5a). Similar to the *in vivo* findings, the LTP deficit *in vitro* was not associated with a change in either baseline AMPA receptor-mediated transmission, determined over the full input–output range (n = 16 WT and 21 TG rats) (Figure 5b), or PPF of these currents (Table 3) in TG animals. Moreover, background AMPA receptor-mediated transmission measured in the absence (spontaneous EPSCs) or presence of the Na^+^ channel blocker tetrodotoxin (miniature EPSCs), was not noticeably different between TG and WT rats. Neither the amplitude nor the frequency of these EPSCs was significantly different between groups (Table 3). Thus, the impairment in LTP in TG rats is unlikely to be caused by any change in spontaneous or evoked synaptic transmission mediated through AMPA receptors.

**Table 3:** **Intrinsic properties, spontaneous and miniature EPSCs, and paired pulse facilitation (PPF) of AMPA receptor-mediated evoked EPSCs were not significantly disrupted in hippocampal slices from pre-plaque 3.5-5 month-old McGill-Thy1-APP TG rats**

	**WT**	**TG**
**Intrinsic Properties**	(n = 7, 9 cells)	(n = 6, 14 cells)
(3.5-5 months)		
Resting membrane	−61.6 ± 2.1	−63.8 ± 1.7
potential (mV)		
τ membrane (ms)	14.3 ± 1.0	15.1 ± 1.0
Sag Ratio	0.9 ± 0.01	0.9 ± 0.01
**sEPSCs**	(n = 5, 6 cells)	(n = 9, 9 cells)
(4–4.5 months)		
Amplitude (pA)	−14.3 ± 1.1	−12.8 ± 1.0
Frequency (Hz)	3.5 ± 1.5	2.4 ± 0.6
**mEPSCs**	(n = 5, 6 cells)	(n = 9, 9 cells)
(4–4.5 months)		
Amplitude (pA)	−12.1 ± 1.1	−11.3 ± 1.1
Frequency (Hz)	1.2 ± 0.4	1.1 ± 0.3
**AMPA EPSCs**	(n = 15, 23 cells)	(n = 25, 44 cells)
(3.5-5 months)		
PPF (ratio) 50 ms IPI	1.4 ± 0.1	1.5 ± 0.04

In order to assess electrically evoked NMDA-receptor mediated component of synaptic transmission we depolarized the membrane to −40 mV and used a stimulation intensity that evoked an AMPA receptor-mediated current of approximately 50% maximum. TG rats had significantly smaller amplitude NMDA receptor-mediated synaptic currents (−102.1 ± 18.6 pA, n = 7; 9 cells; P < 0.05 compared with −166.4 ± 28.7 pA in WT group, n = 7; 9 cells, one-tailed *t* test when grouped by animals, two-tailed when grouped by cells) (Figure 5c). These data are consistent with the *in vivo* finding that the burst response to the conditioning stimulation was reduced, presumably as a result of decreased NMDA receptor-mediated synaptic transmission under depolarizing conditions.

We also measured some intrinsic neurophysiological properties of CA1 pyramidal neurons under current clamp conditions. We found no difference between WT and TG rats (Table 3), indicating that the deficit in NMDA receptor-mediated transmission or LTP was not caused by disruption of these intrinsic properties.

### Relatively rapid-onset persistent inhibition of LTP induction in TG rats followed longitudinally

**Figure 6:**
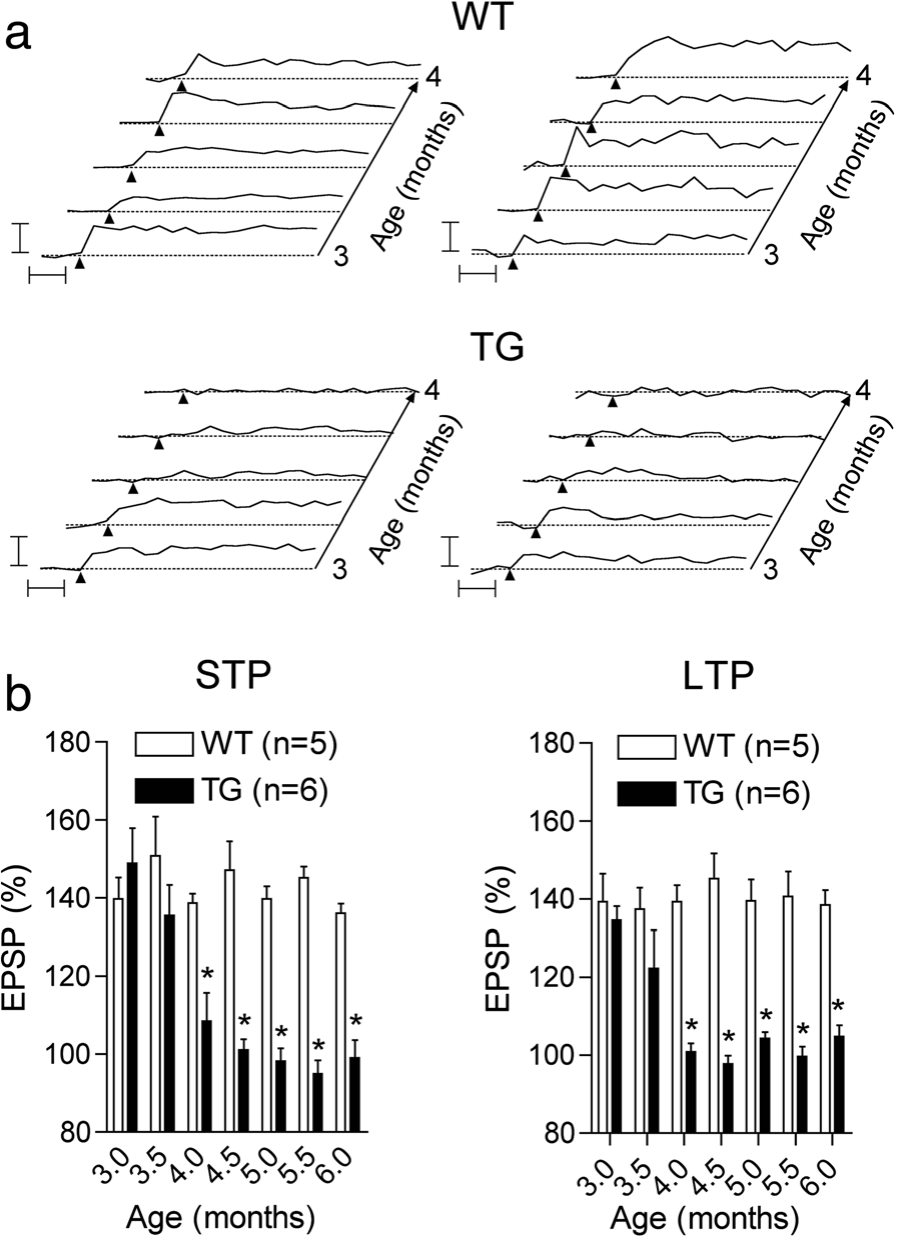
**Longitudinal studies reveal age-dependent relatively rapid-onset and persistent LTP impairment in individual TG rats. (a,b)** The age of onset and time course of the development of the deficit in LTP was assessed using a longitudinal study design. **(a)** We evaluated the ability of the 200 Hz HFS protocol to induce potentiation at weekly intervals in individual animals between 3 and 4 months of age. The top pair of panels shows time-course graphs of LTP induced by repeated weekly application of the HFS protocol in two WT control animals. The HFS triggered similar magnitude LTP each time during the repeated recording sessions. In contrast, the ability to induce STP and LTP was impaired at slightly different ages in individual TG rats as shown in the bottom pair of panels. Arrow heads: HFS application Horizontal lines: 100% baseline EPSP amplitude Calibration bars: Vertical, 50% change in EPSP amplitude; Horizontal, 30 min. **(b)** Summary grouped data for the magnitude of STP and LTP tracked repeatedly in the same WT and TG rats at fortnightly intervals between 3 and 6 months of age. The ability of the HFS protocol to induce LTP was lost between 3 and 4 months of age and the loss persisted for the remaining 2-month recording period. Values in **(b)** are the mean + S.E.M. EPSP amplitude at 3 h after the HFS. *P < 0.05 compared with WT.

Having found that LTP was impaired in 4, but not 3, month-old freely behaving TG rats in the cross-sectional study, we went on to investigate the time course of the deficit in individual animals longitudinally. We were able to study LTP at weekly intervals because the LTP induced by our standard HFS in freely behaving WT rats reverted back to pre-HFS baseline levels within 7 days (data not shown) and LTP was of similar magnitude from week-to-week in WT animals (Figure 6a). The pattern of the time course of the decline in the ability to induce STP and LTP between 3 and 4 months of age varied slightly from rat to rat in the TG group. In the majority of animals the decline appeared to be relatively fast, both STP and LTP apparently disappearing over a 1–2 week period (Figure 6a). In some animals the decline took several weeks, with LTP being more susceptible than STP. In order to determine if the disruption of STP and LTP was stable in individual animals over the months prior to plaque formation we tested them fortnightly up to the age of 6 months. Unlike their WT littermates, from 4 to 6 months of age repeated application of the HFS protocol in individual TG rats consistently failed to trigger significant STP (100.8 ± 7.2 and 99.0 ± 4.6%, n = 6, at 4 and 6 months, respectively; P > 0.05 compared with baseline) or LTP (108.4 ± 2.2 and 104.8 ± 2.9%, n = 6, at 4 and 6 months, respectively; P > 0.05 compared with baseline) (Figure 6b).

### Rapid-onset transient reversal of LTP deficit by agents targeting Aß in TG rats followed longitudinally

Finally, given the maintained inhibition of the ability to induce LTP in TG rats from 4–6 months of age, the question arises as to whether or not the deficit is reversible with therapeutically relevant interventions that are known to target Aß in the brain. In view of the power of the longitudinal design we were particularly interested in profiling the onset and duration of any reversal of the LTP deficit. Because currently available agents can affect non-Aß targets we chose to evaluate the efficacy of three different approaches that differentially affect other components of APP processing: an anti-Aß antibody, a BACE1 inhibitor and a γ-secretase inhibitor.

**Figure 7:**
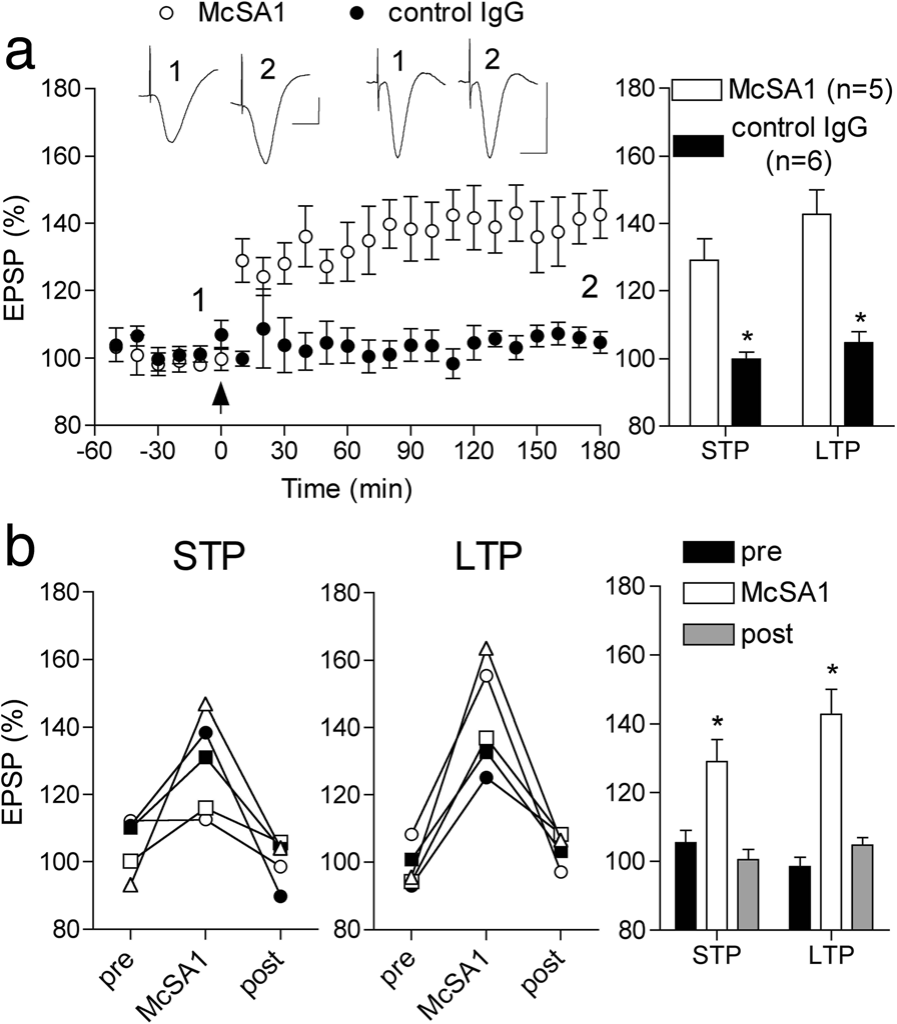
**An anti-Aß antibody rapidly reverses the pre-plaque LTP deficit (a,b).** Three-day treatment with the anti-Aß monoclonal antibody McSA1 transiently restored the ability to induce LTP in 4.5 month-old TG rats. **(a)** In animals treated with McSA1 (5 X 10 μg injections i.c.v.), but not with an isotype control IgG antibody, HFS triggered robust STP and LTP that was stable for at least 3 h. Left hand panel shows the time course of synaptic plasticity. Summary bar chart of STP and LTP in left hand panel. *P < 0.05 compared with isotype control IgG antibody. Insets show representative EPSP traces at the times indicated. Calibration bars: Vertical, 1.0 mV; horizontal, 10 ms. **(b)** The recovery from the impairment in synaptic plasticity lasted for less than two weeks in the McSA1-treated group. The same HFS protocol applied either just before (pre) or two weeks after (post) the injections of McSA1 (n = 5) failed to induce either STP or LTP. Data for individual animals are shown in the first two panels and summarized statistically in the bar charts. *P < 0.05 compared with pre. Values are the mean ± S.E.M. % pre-HFS baseline EPSP amplitude at 10 min (STP) or 3 h (LTP).

We studied the ability to induce LTP 2 h after the last injection (five i.c.v. injections over three days) with these agents. First we assessed McSA1, which is an N-terminally directed monoclonal antibody that recognizes all forms of human Aß [11,16]. TG (4.5 month-old) animals were tracked longitudinally before, during and after receiving the anti-Aß antibody or an IgG isotype control antibody (10 μg per injection). Whereas standard HFS failed to induce LTP prior to McSA1 treatment, the same conditioning protocol triggered LTP in the same TG animals when injected with the antibody. Thus McSA1 restored the ability to induce LTP in TG rats (142.7 ± 7.2%, n = 5; P < 0.05 compared with 104.8 ± 3.2% in animals injected with the control antibody, n = 6; P < 0.05 compared with pre-injection) (Figure 7a). Indeed the magnitude of STP and LTP appeared similar to that induced in age-matched WT rats (see Figure 6b). However, the antibody-mediated reversal of the synaptic plasticity deficit was transient since two weeks after ceasing McSA1 treatment HFS again failed to induce significant STP (100.4 ± 2.9%; P > 0.05 compared with baseline) or LTP in the same TG rats (104.7 ± 2.1%; P > 0.05 compared with baseline) (Figure 7b).

**Figure 8:**
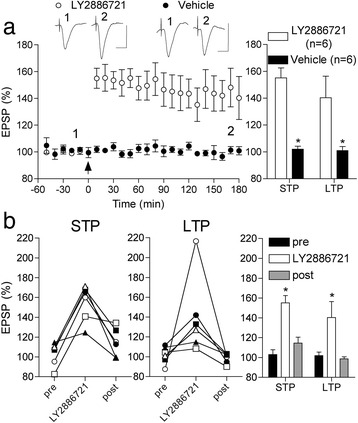
**A BACE1 inhibitor reverses the pre-plaque LTP deficit (a,b).** Three-day treatment with the BACE1 inhibitor LY2886721 transiently restored the ability to induce LTP in 4.5 month-old TG rats. **(a)** In animals treated with LY2886721 (5 X 0.2 nmol injections i.c.v.), but not with vehicle, HFS triggered robust STP and LTP that was stable for at least 3 h. Left hand panel shows the time course of synaptic plasticity. Summary bar chart of STP and LTP in left hand panel. *P < 0.05 compared with vehicle. Insets show representative EPSP traces at the times indicated. Calibration bars: Vertical, 1.0 mV; horizontal, 10 ms. **(b)** The recovery from the impairment in synaptic plasticity lasted for less than a week in the LY2886721-treated group. The same HFS protocol applied either just before (pre) or one week after (post) the injections of LY2886721 (n = 6) failed to induce either STP or LTP. Data for individual animals are shown in the first two panels and summarized statistically in the bar charts. *P < 0.05 compared with pre. Values are the mean ± S.E.M. % pre-HFS baseline EPSP amplitude at 10 min (STP) or 3 h (LTP).

Next we tested the BACE1 inhibitor LY2886721, which is known to reduce cerebral Aß load rapidly [27]. Using a similar protocol to that used when investigating the effect of McSA1, LY2886721 (0.2 nmol per injection i.c.v.) yielded very similar findings in 4.5 month-old TG rats. Thus the pre-treatment LTP deficit in individual animals was reversed by the BACE1 inhibitor (140.3 ± 16.1%, n = 6; P < 0.05 compared with 100.9 ± 3.1% in vehicle-injected TG animals, n = 6; p < 0.05 compared with pre-injection) (Figure 8a). The STP deficit was similarly restored by LY2886721 in TG rats (155.1 ± 7.5%; P < 0.05 compared with 102.0 ± 2.3% in vehicle-injected TG rats) (Figure 8a). This dose of LY2886721 did not affect LTP in WT control animals (136.8 ± 6.2%, n = 3, data not shown; similar in magnitude to age-matched WT animals in the absence of LY2886721, Figure 6b). Again, the reversal of the synaptic plasticity impairment was transient, lasting less than 1 week (Figure 8b).

**Figure 9:**
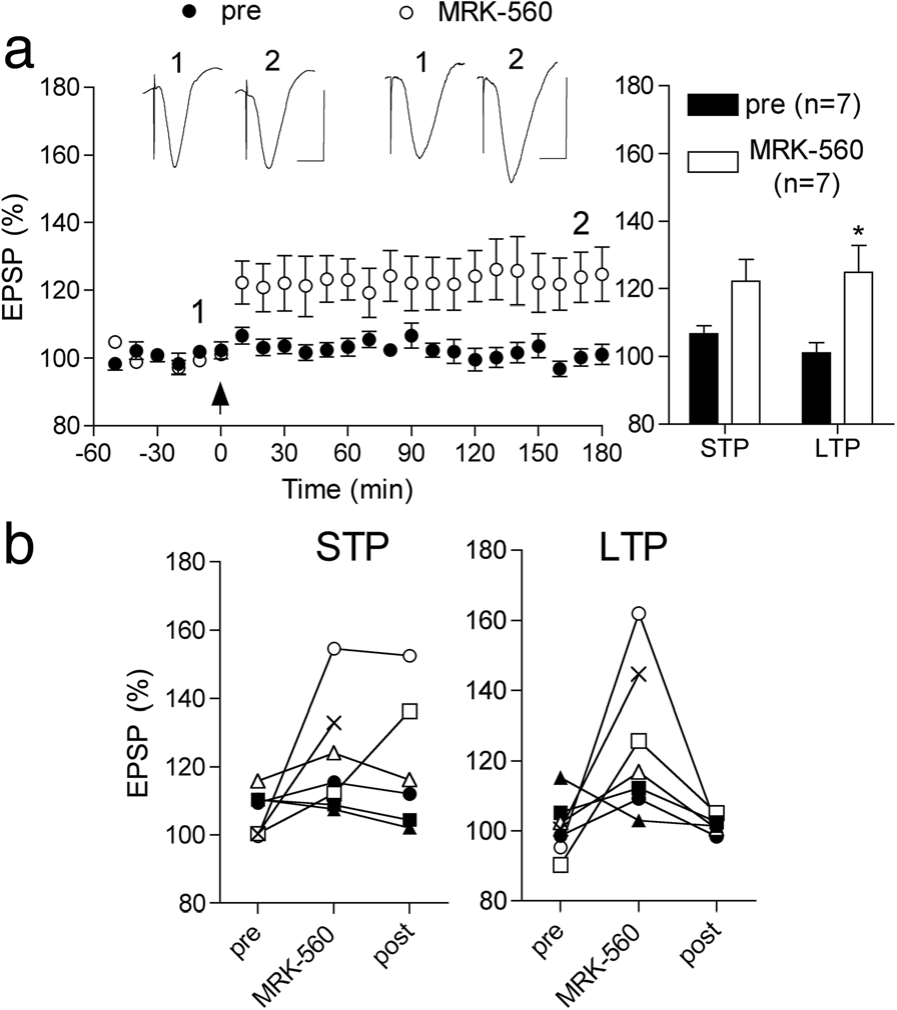
**A γ-secretase inhibitor reverses the pre-plaque LTP deficit (a,b).** Three-day treatment with the γ-secretase inhibitor MRK-560 transiently restored the ability to induce LTP in 5 month-old TG rats. **(a)** In animals treated with MRK-560 (5 X 0.19 nmol injections i.c.v.) HFS triggered robust STP and LTP that was stable for at least 3 h. Left hand panel shows the time course of synaptic plasticity. Summary bar chart of STP and LTP in left hand panel. *P < 0.05 compared with pretreatment levels of potentiation in the same animals (pre). Insets show representative EPSP traces at the times indicated. Calibration bars: Vertical, 1.0 mV; horizontal, 10 ms. **(b)** Data for individual animals pre-, during and one week post-treatment with MRK-560 are shown in the two panels. *P < 0.05 compared with pre. Values are the mean ± S.E.M. % pre-HFS baseline EPSP amplitude at 10 min (STP) or 3 h (LTP).

Finally, treatment with the γ-secretase inhibitor MRK-560 (five i.c.v. injections over 3 days, each injection containing 0.19 nmol) reversed the LTP deficit in 5 month-old animals (124.8 ± 8.0%, n = 7; P < 0.05 compared with 101.1 ± 3.0% in the same animals before starting the drug treatment) (Figure 9). The vehicle was the same as that used in the study with LY2886721 and likewise this dose of MRK-560 did not affect LTP in WT rats (133.8 ± 5.8%, n = 5; P < 0.05 compared with 123.2 ± 3.7% pre-drug treatment, data not shown).

## Discussion

The McGill-R-Thy1-APP TG rat, a very comprehensive model of AD amyloidosis [11], became resistant to the induction of synaptic LTP in the dorsal hippocampus many months prior to the deposition of fibrillar Aß in extracellular plaques. The increase in LTP threshold was accompanied by reductions in (a) STP, (b) the synaptic response to burst stimulation and (c) the NMDA receptor-mediated component of excitatory synaptic transmission. In contrast, there was no detectable change in baseline AMPA receptor-mediated synaptic transmission in pre-plaque animals. Longitudinal studies in freely behaving rats revealed that the LTP deficit was relatively rapid in onset and stable over many months. Importantly, brief treatment with an anti-Aß antibody, a BACE1 inhibitor or a γ-secretase inhibitor transiently reversed the impairment in LTP induction. Overall, these findings strongly indicate that endogenous non-fibrillar human Aß triggers a reversible disruption of synaptic plasticity by impairing synaptic NMDA receptor function very early in the pathogenesis of AD.

In the present study we monitored synaptic transmission and plasticity of that transmission in cross-sectional studies of pre-plaque TG rats and longitudinally in individual animals over several months. We found that synaptic LTP was strongly disrupted at approximately 4 months of age both *in vivo* and *in vitro*: *in vitro* in hippocampal slices and *in vivo* in both acutely anaesthetized and chronically implanted freely behaving TG animals. Importantly, the inhibition of LTP could be overcome by increasing the strength of the conditioning protocol, indicating that the threshold for LTP induction and not LTP maintenance or expression was primarily disrupted at the earliest, pre-plaque stage of amyloidosis. Moreover, the deficit in LTP was relatively selective, baseline synaptic transmission and paired pulse facilitation being unaffected, as measured by field potentials *in vivo* and spontaneous, miniature and evoked AMPA receptor-mediated synaptic currents in slices. A notable feature of the pattern of inhibition of LTP was the accompanying profound and consistent reduction in STP in all three preparations. Moreover, the relatively rapid onset of the disruption of both LTP and STP between 3 and 4 months of age went hand-in-hand in the longitudinal studies.

How the synaptic plasticity deficits described here relate to the known behavioural impairments in hippocampus-dependent tasks of this TG line from the age of 3 months [8,10,11] is uncertain. Whereas learning on a Y-maze was impeded in the TG rats at 6 but not 3 months of age, deficits in spatial reference memory and novel object place learning were detected at 3 months [8,10]. It will be interesting to determine if milder conditioning stimulation protocols than those used here reveal an LTP deficit at 3 months of age.

The impairment of STP led us to suspect that the inhibition of LTP was related to an early event in the process of induction. Because sufficient NMDA receptor activation is critical in initiating both STP and LTP at CA3-to-CA1 synapses with our induction protocols in WT animals [21,24,25] we were encouraged to probe NMDA receptor function in the TG rats *in vivo* by examining the synaptic burst response during conditioning stimulation [22] and *in vitro* by measuring pharmacologically isolated NMDA receptor-mediated synaptic currents recorded intracellularly from pyramidal cells. Consistent with a reduction in NMDA receptor function mediating the block of STP and LTP, we found a reduced synaptic burst response in anaesthetized TG rats that correlated negatively with the magnitude of STP and that was mimicked in WT rats by the application of an NMDA receptor antagonist. Moreover, an ~40% decrease in the NMDA receptor-mediated component of synaptic transmission as observed in slices from TG rats is likely sufficient to increase the threshold for inducing LTP [25,26,28-30]. A reduction in NMDA receptor function at the level of the synapse is therefore likely to mediate the inhibition of both STP and LTP in the TG rats at 4 months of age.

There is a dearth of information regarding *in vivo* pre-plaque neurophysiology in APP TG mice [31-33], possibly because of the well-known technical difficulties in carrying out such experiments in the mouse. Previous *in vitro* reports in pre-plaque APP TG mice have found inconsistent changes in baseline transmission and LTP, the variability being due, at least partly, to technical aspects of using the hippocampal slice preparation [6,34]. So far, the only other APP TG rat model that appears to have been investigated has been reported to have pre-plaque deficits in synaptic LTP in the CA1 area when studied *in vitro* [35]. In a number of TG mouse studies that found deficits in LTP the possible involvement of reduced NMDA receptor-mediated function has been supported [6,36-39]. Thus, for example, in pre-plaque APP_LON_ mice impaired NMDA, and to a lesser extent AMPA, receptor-mediated field potentials were found associated with reduced STP and LTP in the CA1 area [37]. Indeed Cissé et al. [36] posited a critical role for loss of the receptor tyrosine kinase EphB2 in mediating selective reduction in NMDA receptor-mediated synaptic currents in dentate granule cells from pre-plaque APP_SWE/IND_ mice. Genetic restoration of EphB2 not only enhanced NMDA receptor-mediated transmission but also rescued STP and LTP deficits at medial perforant path to granule cell synapses. Furthermore the loss of EphB2 function was triggered by Aß oligomer binding with high affinity to this receptor, which was subsequently internalized. Aß may also downregulate NMDA receptor function via other high affinity binding sites including α7-nicotinic acetylcholine receptors [40,41] and cellular prion protein [42,43]. Somewhat paradoxically, Aß oligomers may reduce synaptic NMDA receptor-mediated synaptic transmission by initially enhancing extrasynaptic currents through NMDA receptors, in particular those containing GluN2B subunits [28,44-46]. Interestingly, we have preliminary evidence that the reduction in NMDA receptor-mediated synaptic transmission in the TG rats was largely due to a decrease in the contribution of GluN2B subunit-containing receptors to the synaptic currents (Harney, S. et al. unpublished).

Consistent with previous research reporting elevated Aß concentration in soluble tris-buffered saline (TBS) brain extracts from pre-plaque TG rats [10] we detected Aß monomer in the membrane and to a lesser extent the extracellular fraction of cortical extracts. However, the levels of SDS stable monomer did not correlate with the magnitude of potentiation in these pre-plaque rats. Although we were unable to definitively detect Aß oligomers using WBs with the antibody 6E10, possibly because of the denaturing conditions in the present study [47,48], evidence supporting the presence of oligomeric intraneuronal Aß has already been provided using super resolution microscopy with the monoclonal antibody Nu1 that selectively recognizes oligomeric conformation [10,11]. Indeed, in pilot experiments we found evidence on tricine gels of hippocampal TBS extracts of a negative correlation between the density of a 6E10 reactive band with a molecular weight of ~12-15 kDa, which corresponds to the C-terminal fragment-ß and possibly Aß trimers [11], and LTP in freely behaving 5–6 month-old TG rats (Wilson, E. N. et al. unpublished). Future studies should revisit this issue using more sensitive and specific assays of soluble Aß oligomers, including ELISAs with a suitable antibody detection system [49]. Rather than rely on correlational analysis, we assessed the role of Aß using three different interventions that share the ability to target Aß in different ways using a combined longitudinal and cross-sectional design. We found that a relatively brief 3-day treatment with these agents transiently reversed the inhibition of LTP in pre-plaque rats. Thus the N-terminus-directed monoclonal antibody McSA1, that directly binds human Aß, and the selective BACE1 inhibitor LY2886721, and γ-secretase inhibitor MRK-560, that both potently inhibit Aß production, all reversed the LTP deficit. These findings are consistent with previous results in cross-sectional studies of pre-plaque TG mice that reported relatively rapid reversal of cognitive impairment by anti-Aß antibodies [50,51], BACE1 inhibitors [52] and γ-secretase inhibitors [53]. Furthermore MRK-560 rapidly restored LTP deficits in pre-plaque TG mouse hippocampus *ex vivo* [34]. Although these interventions separately can affect different non-Aß targets they share the ability to inhibit Aß. Whether or not intracellular or extracellular Aß is the main culprit remains to be determined. Overall, our findings leave little doubt that Aß mediates the impairment in synaptic plasticity in the pre-plaque TG rat.

More sensitive and specific assays may be necessary in order to fully delineate which Aß species is contributing to the deficit in LTP. The relatively rapid on and off rates for the ameliorative effects of treatment with either the anti-Aß antibody or the secretase inhibitors strongly indicates that at this early stage of amyloidosis the synaptic plasticity impairment caused by Aß does not appear to rely on some very slowly developing or irreversible process [4]. Our finding that once an inhibition of LTP was present in a given TG rat the ability to trigger LTP remained compromised for several months prior to plaque deposition will allow future studies to evaluate the persistence of any beneficial effects of continued chronic treatment with these and other potential therapeutic avenues.

## References

[1] Dubois B, Feldman HH, Jacova C, Hampel H, Molinuevo JL, Blennow K, DeKosky ST, Gauthier S, Selkoe D, Bateman R, Cappa S, Crutch S, Engelborghs S, Frisoni GB, Fox NC, Galasko D, Habert MO, Jicha GA, Nordberg A, Pasquier F, Rabinovici G, Robert P, Rowe C, Salloway S, Sarazin M, Epelbaum S, de Souza LC, Vellas B, Visser PJ, Schneider L (2014). Advancing research diagnostic criteria for Alzheimer's disease: the IWG-2 criteria. Lancet Neurol.

[2] Mucke L, Selkoe DJ (2012). Neurotoxicity of amyloid beta-protein: synaptic and network dysfunction. Cold Spring Harb Perspect Med.

[3] Klyubin I, Cullen WK, Hu NW, Rowan MJ (2012). Alzheimer's disease Abeta assemblies mediating rapid disruption of synaptic plasticity and memory. Mol Brain.

[4] Klyubin I, Ondrejcak T, Hayes J, Cullen WK, Mably AJ, Walsh DM, Rowan MJ (2014). Neurotransmitter receptor and time dependence of the synaptic plasticity disrupting actions of Alzheimer's disease Abeta *in vivo*. Philos Trans R Soc Lond Ser B Biol Sci.

[5] Nistico R, Pignatelli M, Piccinin S, Mercuri NB, Collingridge G (2012). Targeting synaptic dysfunction in Alzheimer's disease therapy. Mol Neurobiol.

[6] Randall AD, Witton J, Booth C, Hynes-Allen A, Brown JT (2010). The functional neurophysiology of the amyloid precursor protein (APP) processing pathway. Neuropharmacology.

[7] Do Carmo S, Cuello AC (2013). Modeling Alzheimer's disease in transgenic rats. Mol Neurodegener.

[8] Galeano P, Martino Adami PV, Do Carmo S, Blanco E, Rotondaro C, Capani F, Castano EM, Cuello AC, Morelli L (2014). Longitudinal analysis of the behavioral phenotype in a novel transgenic rat model of early stages of Alzheimer's disease. Front Behav Neurosci.

[9] Hanzel CE, Pichet-Binette A, Pimentel LS, Iulita MF, Allard S, Ducatenzeiler A, Do Carmo S, Cuello AC (2014). Neuronal driven pre-plaque inflammation in a transgenic rat model of Alzheimer's disease. Neurobiol Aging.

[10] Iulita MF, Allard S, Richter L, Munter LM, Ducatenzeiler A, Weise C, Do Carmo S, Klein WL, Multhaup G, Cuello AC (2014). Intracellular Abeta pathology and early cognitive impairments in a transgenic rat overexpressing human amyloid precursor protein: a multidimensional study. Acta Neuropathol Commun.

[11] Leon WC, Canneva F, Partridge V, Allard S, Ferretti MT, DeWilde A, Vercauteren F, Atifeh R, Ducatenzeiler A, Klein W, Szyf M, Alhonen L, Cuello AC (2010). A novel transgenic rat model with a full Alzheimer's-like amyloid pathology displays pre-plaque intracellular amyloid-beta-associated cognitive impairment. J Alzheimers Dis.

[12] Cullen WK, Suh YH, Anwyl R, Rowan MJ (1997). Block of LTP in rat hippocampus *in vivo* by ß-amyloid precursor protein fragments. Neuroreport.

[13] Qi Y, Hu NW, Rowan MJ (2013). Switching off LTP: mGlu and NMDA receptor-dependent novelty exploration-induced depotentiation in the rat hippocampus. Cereb Cortex.

[14] Ryan BK, Vollmayr B, Klyubin I, Gass P, Rowan MJ (2010). Persistent inhibition of hippocampal long-term potentiation *in vivo* by learned helplessness stress. Hippocampus.

[15] Lesne S, Koh MT, Kotilinek L, Kayed R, Glabe CG, Yang A, Gallagher M, Ashe KH (2006). A specific amyloid-beta protein assembly in the brain impairs memory. Nature.

[16] Grant SM, Ducatenzeiler A, Szyf M, Cuello AC (2000). Abeta immunoreactive material is present in several intracellular compartments in transfected, neuronally differentiated, P19 cells expressing the human amyloid beta-protein precursor. J Alzheimers Dis.

[17] Adekar SP, Jones RM, Elias MD, Al-Saleem FH, Root MJ, Simpson LL, Dessain SK (2008). Hybridoma populations enriched for affinity-matured human IgGs yield high-affinity antibodies specific for botulinum neurotoxins. J Immunol Methods.

[18] Fowler SW, Chiang AC, Savjani RR, Larson ME, Sherman MA, Schuler DR, Cirrito JR, Lesne SE, Jankowsky JL (2014). Genetic modulation of soluble Abeta rescues cognitive and synaptic impairment in a mouse model of Alzheimer's disease. J Neurosci.

[19] Handoko M, Grant M, Kuskowski M, Zahs KR, Wallin A, Blennow K, Ashe KH (2013). Correlation of specific amyloid-beta oligomers with tau in cerebrospinal fluid from cognitively normal older adults. JAMA Neurol.

[20] Doyle C, Holscher C, Rowan MJ, Anwyl R (1996). The selective neuronal NO synthase inhibitor 7-nitro-indazole blocks both long-term potentiation and depotentiation of field EPSPs in rat hippocampal CA1 *in vivo*. J Neurosci.

[21] Hu NW, Smith IM, Walsh DM, Rowan MJ (2008). Soluble amyloid-beta peptides potently disrupt hippocampal synaptic plasticity in the absence of cerebrovascular dysfunction *in vivo*. Brain.

[22] Blanpied TA, Berger TW (1992). Characterization *in vivo* of the NMDA receptor-mediated component of dentate granule cell population synaptic responses to perforant path input. Hippocampus.

[23] Grienberger C, Chen X, Konnerth A (2014). NMDA receptor-dependent multidendrite Ca(2+) spikes required for hippocampal burst firing *in vivo*. Neuron.

[24] Anwyl R, Mulkeen D, Rowan MJ (1989). The role of N-methyl-D-aspartate receptors in the generation of short-term potentiation in the rat hippocampus. Brain Res.

[25] Park P, Volianskis A, Sanderson TM, Bortolotto ZA, Jane DE, Zhuo M, Kaang BK, Collingridge GL (2014). NMDA receptor-dependent long-term potentiation comprises a family of temporally overlapping forms of synaptic plasticity that are induced by different patterns of stimulation. Philos Trans R Soc Lond Ser B Biol Sci.

[26] Shipton OA, Paulsen O (2014). GluN2A and GluN2B subunit-containing NMDA receptors in hippocampal plasticity. Philos Trans R Soc Lond Ser B Biol Sci.

[27] May P, Boggs L, Brier R, Calligaro D, Citron M, Day T, Lin S, Lindstrom T, Mergott D, Monk S, Sanchez-Felix MV, Sheehan S, Vaught G, Yang Z, Audia J Preclinical characterization of LY2886721: A BACE1 inhibitor in clinical development for early Alzheimer's disease. Alzheimer's & Dementia: The Journal of the Alzheimer's Association 8: P95 doi10.1016/j.jalz.2012.05.235

[28] Kervern M, Angeli A, Nicole O, Leveille F, Parent B, Villette V, Buisson A, Dutar P (2012). Selective impairment of some forms of synaptic plasticity by oligomeric amyloid-beta peptide in the mouse hippocampus: implication of extrasynaptic NMDA receptors. J Alzheimers Dis.

[29] Raymond CR, Ireland DR, Abraham WC (2003). NMDA receptor regulation by amyloid-beta does not account for its inhibition of LTP in rat hippocampus. Brain Res.

[30] Ryan BK, Anwyl R, Rowan MJ (2008). 5-HT2 receptor-mediated reversal of the inhibition of hippocampal long-term potentiation by acute inescapable stress. Neuropharmacology.

[31] Davis KE, Fox S, Gigg J (2014). Increased hippocampal excitability in the 3xTgAD mouse model for Alzheimer's disease *in vivo*. PLoS One.

[32] Gruart A, Lopez-Ramos JC, Munoz MD, Delgado-Garcia JM (2008). Aged wild-type and APP, PS1, and APP + PS1 mice present similar deficits in associative learning and synaptic plasticity independent of amyloid load. Neurobiol Dis.

[33] Mitchell JC, Ariff BB, Yates DM, Lau KF, Perkinton MS, Rogelj B, Stephenson JD, Miller CC, McLoughlin DM (2009). X11beta rescues memory and long-term potentiation deficits in Alzheimer's disease APPswe Tg2576 mice. Hum Mol Genet.

[34] Townsend M, Qu Y, Gray A, Wu Z, Seto T, Hutton M, Shearman MS, Middleton RE (2010). Oral treatment with a gamma-secretase inhibitor improves long-term potentiation in a mouse model of Alzheimer's disease. J Pharmacol Exp Ther.

[35] Liu L, Orozco IJ, Planel E, Wen Y, Bretteville A, Krishnamurthy P, Wang L, Herman M, Figueroa H, Yu WH, Arancio O, Duff K (2008). A transgenic rat that develops Alzheimer's disease-like amyloid pathology, deficits in synaptic plasticity and cognitive impairment. Neurobiol Dis.

[36] Cisse M, Halabisky B, Harris J, Devidze N, Dubal DB, Sun B, Orr A, Lotz G, Kim DH, Hamto P, Ho K, Yu GQ, Mucke L (2011). Reversing EphB2 depletion rescues cognitive functions in Alzheimer model. Nature.

[37] Dewachter I, Filipkowski RK, Priller C, Ris L, Neyton J, Croes S, Terwel D, Gysemans M, Devijver H, Borghgraef P, Godaux E, Kaczmarek L, Herms J, Van Leuven F (2009). Deregulation of NMDA-receptor function and down-stream signaling in APP[V717I] transgenic mice. Neurobiol Aging.

[38] Tozzi A, Sclip A, Tantucci M, de Iure A, Ghiglieri V, Costa C, Di Filippo M, Borsello T, Calabresi P (2014) Region- and age-dependent reductions of hippocampal long-term potentiation and NMDA to AMPA ratio in a genetic model of Alzheimer's disease. Neurobiology of aging: doi10.1016/j.neurobiolaging.2014.07.002 10.1016/j.neurobiolaging.2014.07.002 25104560

[39] Wang Y, Mattson MP (2014). L-type Ca2+ currents at CA1 synapses, but not CA3 or dentate granule neuron synapses, are increased in 3xTgAD mice in an age-dependent manner. Neurobiol Aging.

[40] Snyder EM, Nong Y, Almeida CG, Paul S, Moran T, Choi EY, Nairn AC, Salter MW, Lombroso PJ, Gouras GK, Greengard P (2005). Regulation of NMDA receptor trafficking by amyloid-beta. Nat Neurosci.

[41] Wang HY, Lee DH, D'Andrea MR, Peterson PA, Shank RP, Reitz AB (2000). beta-Amyloid(1–42) binds to alpha7 nicotinic acetylcholine receptor with high affinity. Implications for Alzheimer's disease pathology. J Biol Chem.

[42] Lauren J, Gimbel DA, Nygaard HB, Gilbert JW, Strittmatter SM (2009). Cellular prion protein mediates impairment of synaptic plasticity by amyloid-beta oligomers. Nature.

[43] Um JW, Nygaard HB, Heiss JK, Kostylev MA, Stagi M, Vortmeyer A, Wisniewski T, Gunther EC, Strittmatter SM (2012). Alzheimer amyloid-beta oligomer bound to postsynaptic prion protein activates Fyn to impair neurons. Nat Neurosci.

[44] Hu NW, Klyubin I, Anwyl R, Rowan MJ (2009). GluN2B subunit-containing NMDA receptor antagonists prevent Abeta-mediated synaptic plasticity disruption *in vivo*. Proc Natl Acad Sci U S A.

[45] Li S, Jin M, Koeglsperger T, Shepardson NE, Shankar GM, Selkoe DJ (2011). Soluble Abeta oligomers inhibit long-term potentiation through a mechanism involving excessive activation of extrasynaptic NR2B-containing NMDA receptors. J Neurosci.

[46] Ronicke R, Mikhaylova M, Ronicke S, Meinhardt J, Schroder UH, Fandrich M, Reiser G, Kreutz MR, Reymann KG (2011). Early neuronal dysfunction by amyloid beta oligomers depends on activation of NR2B-containing NMDA receptors. Neurobiol Aging.

[47] Lesne SE, Sherman MA, Grant M, Kuskowski M, Schneider JA, Bennett DA, Ashe KH (2013). Brain amyloid-beta oligomers in ageing and Alzheimer's disease. Brain.

[48] Melnikova T, Fromholt S, Kim H, Lee D, Xu G, Price A, Moore BD, Golde TE, Felsenstein KM, Savonenko A, Borchelt DR (2013). Reversible pathologic and cognitive phenotypes in an inducible model of Alzheimer-amyloidosis. J Neurosci.

[49] Yang T, Hong S, O'Malley T, Sperling RA, Walsh DM, Selkoe DJ (2013). New ELISAs with high specificity for soluble oligomers of amyloid beta-protein detect natural Abeta oligomers in human brain but not CSF. Alzheimers Dement.

[50] Dodart JC, Bales KR, Gannon KS, Greene SJ, DeMattos RB, Mathis C, DeLong CA, Wu S, Wu X, Holtzman DM, Paul SM (2002). Immunization reverses memory deficits without reducing brain Abeta burden in Alzheimer's disease model. Nat Neurosci.

[51] Kotilinek LA, Bacskai B, Westerman M, Kawarabayashi T, Younkin L, Hyman BT, Younkin S, Ashe KH (2002). Reversible memory loss in a mouse transgenic model of Alzheimer's disease. J Neurosci.

[52] Fukumoto H, Takahashi H, Tarui N, Matsui J, Tomita T, Hirode M, Sagayama M, Maeda R, Kawamoto M, Hirai K, Terauchi J, Sakura Y, Kakihana M, Kato K, Iwatsubo T, Miyamoto M (2010). A noncompetitive BACE1 inhibitor TAK-070 ameliorates Abeta pathology and behavioral deficits in a mouse model of Alzheimer's disease. J Neurosci.

[53] Comery TA, Martone RL, Aschmies S, Atchison KP, Diamantidis G, Gong X, Zhou H, Kreft AF, Pangalos MN, Sonnenberg-Reines J, Jacobsen JS, Marquis KL (2005). Acute gamma-secretase inhibition improves contextual fear conditioning in the Tg2576 mouse model of Alzheimer's disease. J Neurosci.

